# Genomic Survey and Microsatellite Marker Investigation of Patagonian Moray Cod (*Muraenolepis orangiensis*)

**DOI:** 10.3390/ani12131608

**Published:** 2022-06-22

**Authors:** Eunkyung Choi, Seung Jae Lee, Euna Jo, Jinmu Kim, Steven J. Parker, Jeong-Hoon Kim, Hyun Park

**Affiliations:** 1 Division of Biotechnology, College of Life Sciences and Biotechnology, Korea University, Seoul 02841, Korea; amy_choi@korea.ac.kr (E.C.); skullcap@korea.ac.kr (S.J.L.); eunajo@kopri.re.kr (E.J.); rlawlsan04@korea.ac.kr (J.K.); 2 Korea Polar Research Institute (KOPRI), Incheon 21990, Korea; jhkim94@kopri.re.kr; 3 Commission for the Conservation of Antarctic Marine Living Resources (CCAMLR), Hobart, TAS 7000, Australia; steve.parker@ccamlr.org

**Keywords:** *Muraenolepis orangiensis*, Patagonian moray cod, microsatellite, SSR, Illumina

## Abstract

**Simple Summary:**

Patagonian moray cod is known to inhabit the cold waters near Antarctica, and it belongs to the Muraenolepis genus. This genus has seven species, and five of them are recently reported. The Muraenolepis genus has similar morphological characters, and this is a limitation of taxonomical classification. In this study, a genome survey and microsatellite marker analysis were conducted to characterize the genome profile for classification. As a result, genomic data such as genome size and microsatellite motifs were obtained.

**Abstract:**

The Muraenolepididae family of fishes, known as eel cods, inhabits continental slopes and shelves in the Southern Hemisphere. This family belongs to the Gadiformes order, which constitutes one of the most important commercial fish resources worldwide, but the classification of the fish species in this order is ambiguous because it is only based on the morphological and habitat characteristics of the fishes. Here, the genome of Patagonian moray cod was sequenced using the Illumina HiSeq platform, and screened for microsatellite motifs. The genome was predicted to be 748.97 Mb, with a heterozygosity rate of 0.768%, via K-mer analysis (K = 25). The genome assembly showed that the total size of scaffolds was 711.92 Mb and the N50 scaffold length was 1522 bp. Additionally, 4,447,517 microsatellite motifs were identified from the genome survey assembly, and the most abundant motif type was found to be AC/GT. In summary, these data may facilitate the identification of molecular markers in Patagonian moray cod, which would be a good basis for further whole-genome sequencing with long read sequencing technology and chromosome conformation capture technology, as well as population genetics.

## 1. Introduction

The Muraenolepididae family of fishes, known as eel cods, belongs to the Gadiformes order and inhabits the cold–temperate waters of continental slopes and shelves in the Southern Hemisphere. They dwell near the bottom of the ocean, at the midwater depth, or near the surface. The family has the following morphological characteristics: the dorsal, anal, and caudal fins are all joined; the chin has a barbel; and there are no teeth on the head of some. The family consists of two genera—Notomuraenobathys (Balushkin and Pirodina, 2010) and Muraenolepis (Günther, 1880) [1,2].

The genus of Muraenolepis is regularly caught around South Georgia and the South Sandwich Islands [3], and has been reported to be preyed on by the Patagonian toothfish (*Dissostichus eleginoides*) [4]. Muraenolepis comprises seven species, and five of these species have been mostly described recently (*Muraenolepis marmorata*, Günther 1880; Patagonian moray cod, Vaillant 1888; *Muraenolepis andriashevi*, Balushkin & Prirodina 2005; *Muraenolepis trunovi*, Balushkin & Prirodina 2006; *Muraenolepis kuderskii*, Balushkin & Prirodina 2007; *Muraenolepis pacifica*, Prirodina & Balushkin 2007; and *Muraenolepis evseenkoi*, Balushkin & Prirodina 2010) [5,6,7,8,9,10].

Gadiformes fishes, such as cod and hake, are among the most important commercial fishes worldwide. Despite their commercial importance, the taxonomic classification of these fishes is still far from clear, because it is based on morphological data [11]. Likewise, Muraenolepis fishes have been classified based on their morphological and habitat characteristics; various views have been reported taxonomically, including Gadiformes [5,12].

To overcome the limitations of the morphology-based taxonomic classification of Muraenolepis fishes, a study has analyzed the mitochondrial 16S and COI sequences of these fish species, and thereby confirmed that *Muraenolepis microps* is a junior synonym of *M. marmorata* [13]. Morphological characteristics are taxonomically very important. However, there are species that are difficult to classify accurately based on morphological characteristics alone, so genome-level information is needed to supplement this. In particular, the applicability of microsatellites (also known as simple sequence repeats (SSRs) in differentiating between fish species has been validated [14], and the complete mitochondrial genome of a Muraenolepis fish species has been sequenced [15].

In this study, we assessed the genomic characteristics of Patagonian moray cod via a genomic survey based on next-generation sequencing (NGS) and then identified SSRs that can be used as markers for taxonomic classification. The data from this study may facilitate further genomic characterization of Patagonian moray cod.

## 2. Materials and Methods

### 2.1. Sample Preparation and Sequencing

A sample was collected from the Ross Sea (77°05′ S, 170°30′ E on CCAMLR Subarea 88.1), Antarctica, and then transferred to a freezer at −80 °C. The muscle tissue was dissected from the frozen sample for DNA extraction. The traditional phenol–chloroform method was conducted to obtain the DNA. The quality and quantity of the DNA were assessed using a fragment analyzer (Agilent Technologies, Santa Clara, CA, USA) and a Qubit 2.0 Fluorometer (Life Technologies, Carlsbad, CA, USA), respectively. Then, 1 µg DNA was sheared into 350 bp fragments, using a Covaris S2 system (Covaris, Woburn, MA, USA). DNA library preparation was performed using TruSeq DNA PCR-Free (Illumina, San Diego, CA, USA) according to the manufacturer’s protocol. The library quality was assessed using a Bioanalyzer (Agilent Technologies, Santa Clara, CA, USA), and then the Illumina Novaseq 6000 platform with 2 × 150 bp format (Illumina, San Diego, CA, USA) was used for sequencing.

### 2.2. Data Analysis for Genome Survey and Microsatellite Identification

The quality (Q) values (Q20 and Q30) were estimated from the primary data using seqtk version 1.3 (Available online: https://github.com/lh3/seqtk (accessed on 20 January 2020). All of the clean reads were used to conduct the K-mer analysis to estimate the genome size. Jellyfish version 2.1.4 was used for the K-mer analysis with K-values of 17, 19, and 25. Based on the K-mer data, GenomeScope was used to determine the genome size, repeat contents, and heterozygosity rate [16,17]. The draft de novo genome assembly was carried out using Maryland Super-Read Celera Assembler (MaSuRCA version 3.3.4) [18]. For the genome-wide microsatellite investigation, the QDD pipeline (version 3.1.2) [19] was used. The microsatellite repeats were investigated for their lengths and nucleotide repeats (from mononucleotide repeats to hexanucleotide repeats). The analysis was conducted in three steps, with the parameters of -contig 1, -make_cons 0, and -contig 1. The final output was used to select the best primer pairs for the microsatellite repeats, and a total of 83 primer pairs were selected according to the following parameters of the QDD pipeline (version 3.1.2): forward and reverse flanking regions between the SSR and the primer sequences ≥ 20 bp, a maximum primer alignment score of 5, the high-quality primer design defined by the QDD pipeline, and ≥7 motif repeats [20]. Among these primer pairs, 25 pairs were randomly selected for validation via PCR, performed in a Thermal Cycler Dice^®^ Touch (Takara Bio, Shiga, Japan). Each PCR tube (20 µL volume) contained 5 µL genomic DNA (20 ng/µL), 1 µL (10 pmole/L) of each forward and reverse primer, 10 µL of 2 × EmeraldAmp PCR Master Mix (Takara Bio, Kusatsu, Japan), and 3 µL of double-distilled water. The PCR conditions were as follows: 2 min at 94 °C, followed by 35 cycles of 94 °C for 30 s, 60 °C for 30 s, and 70 °C for 1 min, and the last extension at 72 °C for 10 min. The PCR products were visualized via 4% agarose gel electrophoresis with a 20 bp DNA ladder (Takara Bio, Kusatsu, Japan).

## 3. Results and Discussion

### 3.1. Genome Size Estimation and Genome Assembly

**Figure 1:**
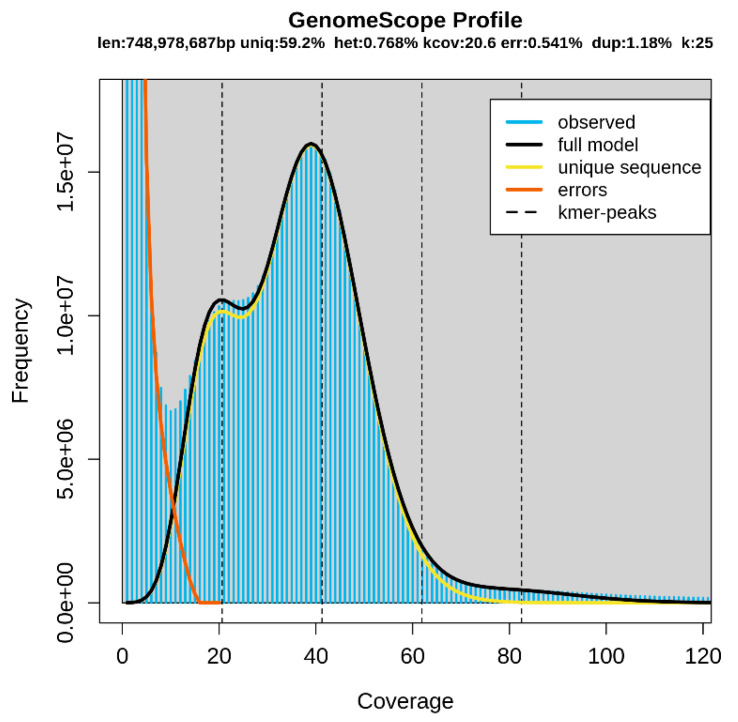
K-mer analysis (K = 25).

A total of 54.14 Gb of raw data were generated using the paired-end library method in the Illumina NovaSeq platform. The Q20 and Q30 values were 93.3% and 87.2%, respectively (Table 1). Q20 and Q30 values ≥ 90% and ≥85%, respectively, indicate accurate sequencing [21]. Therefore, the accuracy of sequencing for the Patagonian moray cod was high. In addition, the GC content was 49.5% (Table 1). These data were used to estimate the genome size via K-mer analysis. Based on the 25-mer distribution, the predicted genome size was approximately 748.9 Mb, and the heterozygous rate was estimated at 0.768%. The duplication rate was 1.18%, and the highest frequency was nearly 40× coverage (Table 2 and Figure 1). MaSuRCA was used to perform a genome assembly (Table 3). We obtained 661,719 scaffolds, and the total size of the scaffolds was 711,920,928 bp. The longest scaffold size was 67,330 bp, and the number of scaffolds longer than 1 Kb was 211,863. The N50 scaffold length was 1522 bp, and the GC content was 45.7%. These genome survey results can serve as preliminary data for further whole-genome studies with advanced sequencing technologies using long read sequencing technology and chromosome conformation capture technology to achieve a more thorough assembly.

Len, estimated total genome length; Uniq, unique portion of the genome (not repetitive); Het, heterozygosity rate; Kcov, mean K-mer coverage for the heterozygous bases; Err, error rate; Dup, duplication rate. The blue bars and black line show the observed K-mer distribution and the modeled distribution without the error K-mers (indicated by the red line), respectively, up to a maximum K-mer coverage specified in the model (indicated by the yellow line).

### 3.2. Identification of Microsatellite Motifs

**Figure 2:**
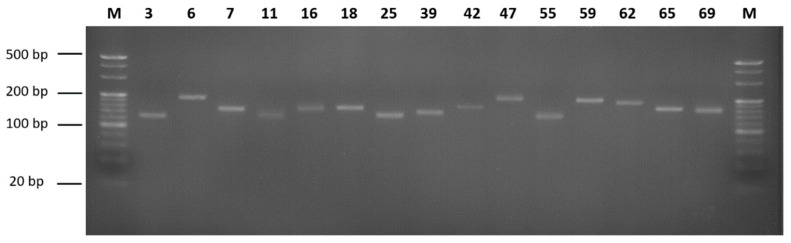
PCR products corresponding to the microsatellites. M is the 20 bp DNA marker and 4% agarose gel electrophoresis was used. The number is primer pairs order.

A total of 4,447,517 microsatellite motifs were identified from the genome survey assembly of Patagonian moray cod. The types of the motifs were dinucleotide, trinucleotide, tetranucleotide, and pentanucleotide, with the fractions of 77.40% (3,430,720), 17.89% (793,122), 3.97% (175,791), and 0.74% (21,801), respectively (Table 4). The most frequent dinucleotide motif was found to be AC/GT (61.21%), followed by AG/CT (24.80%), AT/AT (13.83%), and CG/CG (0.16%). The most abundant trinucleotide motif was found to be AGG/CCT (29.04%), followed by AAT/ATT (18.99%), ACC/GGT (14.34%), AAC/GTT (9.62%), AAG/CTT (9.03%), ATC/GAT (6.49%), AGC/GCT (5.24%), ACT/AGT (3.89%), CCG/CGG (2.69%), and ACG/CGT (0.67%). Regarding the tetranucleotide and pentanucleotide motifs, the most frequent motifs were ACAG/CTGT (27.78%) and AGAGG/CCTCT (32.37%), respectively (Table 4). To compare the microsatellite motifs of Patagonian moray cod with other species, the microsatellite motif analysis results of *Trematomus loennbergii* and *Pogonophryne albipinna* were used [22,23]. When comparing the number of microsatellites, Patagonian moray cod had the most SSRs, and there was no significant difference in the number of SSRs between *T. loennbergii* and *P. albipinna*. When comparing the repeat type, it was confirmed that the ratio of trinucleotides was slightly higher in Patagonian moray cod than in other species. Additionally, unlike other species, Patagonian moray cod did not have a hexanucleotide type (Table S1). Based on the microsatellite motif analysis and the parameters of the QDD pipeline, 83 primer pairs were selected (Table S2). To ensure the usability of these microsatellite markers, 25 primer pairs were randomly selected and used for PCR with the genomic DNA of Patagonian moray cod. Consequently, 15 primer pairs were observed to yield only a single band (Figure 2).

With the advance of NGS technology and bioinformatics tools, genome survey sequencing and K-mer analysis have developed rapidly, assisting with the prediction of genome size and characteristics in non-model species without basic genomic information. According to a flow cytometry-based approach, previous studies have estimated that the genome sizes of several species living in Antarctica were 0.7–1.4 Gb [24]. Recent studies using NGS technology estimated the genome sizes of Antarctic fishes at 0.6–1 Gb [25,26,27,28,29]. In this study, the genome size of Patagonian moray cod was 0.7 Gb and it was within the range of the previously reported genome sizes of Antarctic fishes. Therefore, the genome size of Patagonian moray cod is an acceptable result. For the genome assembly, if the heterozygosity rate is more than 0.5%, it is difficult to assemble. The heterozygosity rate of Patagonian moray cod was 0.7%. It might have been affected by the use of short scaffolds based on short read sequencing technology. In addition, the GC content is also one of the factors affecting sequence bias; if the GC content is more than 65% or less than 25%, sequence bias may influence the genome assembly quality [30,31,32]. The GC content of Patagonian moray cod was 45.7%, and this was within the range from 25% to 65%, which was a normal range. Therefore, the assembly result of this study might be of acceptable range, and the GC content of Patagonian moray cod would be not affected by genome sequence quality. This is the first report of a genome survey of Patagonian moray cod, and these data provide a preliminary understanding of the genome characteristics of Patagonian moray cod. However, a further study with long read sequencing technology and chromosomal-level scaffolding technology is needed to obtain more high-quality genome information for Patagonian moray cod. For identifying the microsatellite markers, using genome survey data has cost- and time-effective advantages compared with the traditional microsatellite marker development method. In this study, the most frequent motifs were dinucleotide repeat motifs, except for the mononucleotide repeat motif, and the less abundant dinucleotide repeat motifs were CG/CG. This result was comparable with other previous studies on microsatellite repeats using *Danio rerio*, *Oreochromis latipes*, and *O. niloticus* [33], and this may be because cytosine is methylated with thymidine [34]. The tendency of the overall motif frequency was quite similar to other studies with fishes [35,36]. This data may be useful in identifying the microsatellite markers of Patagonian moray cod. Moreover, if further studies using various Muraenolepis populations are conducted, more meaningful data for molecular marker development would be obtained.

## 4. Conclusions

In the present study, the genome of Patagonian moray cod was assembled, and the microsatellite motifs were characterized. Therefore, the genome size of Patagonian moray cod was estimated at 748.9 Mb, based on the K-mer analysis. In addition, SSR analysis yielded 4,447,517 SSRs, and the most abundant repeat motif was found to be a dinucleotide, with the most frequent dinucleotide motif of AC/GT.

These genomic data may be useful for verifying taxonomical classifications, and it would be basic information for developing novel molecular markers in various populations. However, further studies, such as a more complete genome assembly at the chromosomal level, and validation experiments using various Muraenolepis populations, would be needed.

## Figures and Tables

**Table 1:** Statistics of the genome-sequencing data.

Raw Data (bp)	Q20 (%)	Q30 (%)	GC Content (%)
54,142,458,226	93.3	87.2	49.5

**Table 2:** Genome size estimation via K-mer analysis.

	Genome Size (bp)	Heterozygosity (%)	Duplication Ratio (%)
17-mer	709,066,708	0.82	1.3
19-mer	723,179,522	0.832	1.23
25-mer	748,978,687	0.768	1.18

**Table 3:** Statistics of the assembled genomic sequences.

	MaSuRCA
Number of scaffolds	661,719
Total size of scaffolds	711,920,928
Longest scaffolds	67,330
Number of scaffolds > 1K nt	211,863
Number of scaffolds > 10K nt	1199
N50 scaffold length	1522
L50 scaffold count	120,833
GC content (%)	45.7

**Table 4:** Distribution of the microsatellite motifs.

Repeat Motif	Number of Repeats								
	5	6	7	8	9	10	11–20	>20	Total
**Dinucleotide (3,430,720)**									
AC/GT	329,788	224,159	178,326	154,725	144,969	134,928	710,095	223,051	2,100,041
AG/CT	230,644	141,955	96,287	67,788	50,979	39,120	158,771	65,210	850,754
AT/AT	119,678	76,695	51,221	38,042	31,331	24,631	122,875	10,068	474,541
CG/CG	3286	1359	427	156	141	15	0	0	5384
**Trinucleotide (793,122)**									
AGG/CCT	64,598	41,234	30,104	21,653	15,244	10,409	42,322	4735	230,299
AAT/ATT	40,273	26,132	19,051	14,810	12,857	9999	26,160	1321	150,603
ACC/GGT	37,009	28,553	19,322	12,185	6659	4288	5583	102	113,701
AAC/GTT	31,660	18,564	11,204	5687	3476	1592	3708	404	76,295
AAG/CTT	18,427	12,484	7928	6032	4720	3360	11,275	7388	71,614
ATC/GAT	14,451	10,771	6815	4771	3575	2427	7904	795	51,509
AGC/GCT	16,945	9439	5433	3676	2141	1205	2433	324	41,596
ACT/AGT	9510	6022	4239	2686	2189	1570	4066	564	30,846
CCG/CGG	10,871	5428	2073	1244	695	401	616	0	21,328
ACG/CGT	2637	1513	640	252	123	53	113	0	5331
**Tetranucleotide (175,791)**									
ACAG/CTGT	12,245	8574	5599	3472	3155	2310	7692	510	43,557
AGGG/CCCT	10,965	6691	4420	2849	2176	1828	1754	0	30,683
ACGC/GCGT	1952	1653	885	758	607	757	3761	786	11,159
AAAG/CTTT	2532	1485	1131	527	407	380	2587	802	9851
AAAC/GTTT	3692	2538	1319	861	371	258	254	0	9293
AAAT/ATTT	3812	1907	822	476	285	98	520	89	8009
ACTC/GAGT	1846	1499	853	459	319	828	1834	215	7853
ACAT/ATGT	2236	1207	920	565	375	481	1481	171	7436
AAGG/CCTT	2474	1144	607	304	178	136	655	242	5740
ATCC/GGAT	2643	1129	527	344	110	128	473	77	5431
Others	14,624	7669	4304	2517	1748	1238	4132	547	36,779
**Pentanucleotide (32,801)**									
AGAGG/CCTCT	2090	1072	733	467	606	365	2378	33	7744
AATGT/ACATT	976	554	333	225	82	12	9	0	2191
AATCT/AGATT	1044	392	244	183	72	42	9	0	1986
AACAC/GTGTT	897	358	270	149	69	15	45	0	1803
AGGGG/CCCCT	968	419	161	113	15		0	0	1676
AATAT/ATATT	431	319	201	116	129	60	180	0	1436
Others	2932	1735	739	694	268	212	509	0	7089
**Total**	**1,008,087**	**649,482**	**459,698**	**350,482**	**291,381**	**243,972**	**1,126,900**	**317,515**	**4,447,517**

## Data Availability

The Patagonian moray cod genome project was registered in NCBI (Bioproject number: PRJNA802969). The whole-genome sequencing data were deposited in the Sequence Read Archive database (accession number: SRR17897366).

## Supplementary Materials

The following supporting information can be downloaded at: https://www.mdpi.com/article/10.3390/ani12131608/s1. Table S1: Number and percentage of di- to hexanucleotide repeats in Antarctic fish; Table S2: List of SSR primers of Patagonian moray cod.
