# Supplementary material for: Genomic Survey and Microsatellite Marker Investigation of Patagonian Moray Cod (Muraenolepis orangiensis)
Source: Animals (Basel). 2022 Jun 22;12(13):1608. doi: 10.3390/ani12131608 (PMC9265078; doi:10.3390/ani12131608)
Supplement: Supplementary file 1 [file animals-12-01608-s001.zip › Table S1_Number and percentage of di- to hexanucleotide repeats in Antarctic fish.pdf]

Table S1. Number and percentage of di- to hexanucleotide repeats in Antarctic fish

Repeat Type	Parameter	*M. orangiensis*	*T. loennbergii*	*P. albipinna*
Di-	Number of SSRs	3,430,720	1,970,270	1,926,231
	Percentage (%)	77.14	87.00	86.87
Tri-	Number of SSRs	793,122	236,541	249,028
	Percentage (%)	17.83	10.44	11.23
Tetra-	Number of SSRs	175,791	4,3907	36,955
	Percentage (%)	3.95	1.93	1.67
Penta-	Number of SSRs	32,801	7,733	3,372
	Percentage (%)	0.74	0.34	0.15
Hexa-	Number of SSRs	-	6,196	1,836
	Percentage (%)	-	0.27	0.08
